# Dynamics of adrenergic signaling in cardiac myocytes and implications for pharmacological treatment

**DOI:** 10.1016/j.jtbi.2021.110619

**Published:** 2021-03-16

**Authors:** Emily E. Meyer, Colleen E. Clancy, Timothy J. Lewis

**Affiliations:** University of California Davis, Davis, CA, United States

**Keywords:** Sympathetic nervous system, Cyclic AMP, Heart failure, *β*-blockers, Mathematical model

## Abstract

Dense innervation of the heart by the sympathetic nervous system (SNS) allows cardiac output to respond appropriately to the needs of the body under varying conditions, but occasionally the abrupt onset of SNS activity can trigger cardiac arrhythmias. Sympathetic activity leads to the release of norepinephrine (NE) onto cardiomyocytes, activating *β*_1_-adrenergic receptors (*β*_1_-ARs) and leading to the production of the second messenger cyclic AMP (cAMP). Upon sudden activation of *β*_1_-ARs in experiments, intracellular cAMP can transiently rise to a high concentration before converging to a steady state level. Although changes to cellular cAMP concentration are important in modulating the overall cardiovascular response to sympathetic tone, the underlying mechanisms of the cAMP transients and the parameters that control their magnitude are unclear.

We reduce a detailed computational model of the *β*_1_-adrenergic signaling cascade to a system of two differential equations by eliminating extraneous variables and applying quasi-steady state approximation. The structure of the reduced model reveals that the large cAMP transients associated with abrupt *β*_1_-AR activation are generated by the interplay of production/degradation of cAMP and desensitization/resensitization of *β*_1_-ARs. The reduced model is used to predict how the dynamics of intracellular cAMP depend on the concentrations of norepinephrine (NE), phosphodiesterases 3 and 4 (PDE3,4), G-protein coupled receptor kinase 2 (GRK2), and *β*_1_-AR, in healthy conditions and a simple model of early stages of heart failure.

The key findings of the study are as follows: 1) Applying a reduced model of the dynamics of cardiac sympathetic signaling we show that the concentrations of two variables, cAMP and non-desensitized *β*_1_-AR, capture the overall dynamics of sympathetic signaling; 2) The key factors influencing cAMP production are AC activity and PDE3,4 activity, while those that directly impact *β*_1_-AR phosphorylation are GRK2 and PKA_1_. Thus, disease states that affect sympathetic control of the heart can be thoroughly assessed by studying AC activity, PDE3,4, GRK2 and PKA activity, as these factors directly impact cAMP production/degradation and *β*_1_-AR (de) phosphorylation and are therefore predicted to comprise the most effective pharmaceutical targets in diseases affecting cardiac *β*_1_-adrenergic signaling.

## 1. Introduction

Activity of the sympathetic nervous system (SNS) modulates overall cardiovascular function: in healthy mammals, heart rate and contractile force adapt dynamically in response to sympathetic activity. However, dysregulation of the SNS has been linked to proarrhythmia and heart failure (Florea and Cohn, 2014; Lymperopoulos et al., 2013; Tomaselli and Zipes, 2004; Ripplinger et al., 2016). Enhanced sympathetic activity is associated with exacerbation of prior heart failure and sudden cardiac death (Brunner-La Rocca et al., 2001; Nishimura et al., 2010). The changes resulting from the onset of SNS activity require cardiomyocytes to increase their production of the second messenger cyclic AMP (cAMP), which suggests that cAMP is an important component of the cardiac response to the SNS in physiological and pathological conditions. It is therefore critical to understand the dynamical mechanisms of adrenergic signaling in cardiac myocytes and how this signaling modulates cellular cAMP levels in health and disease.

**Fig. 1.**
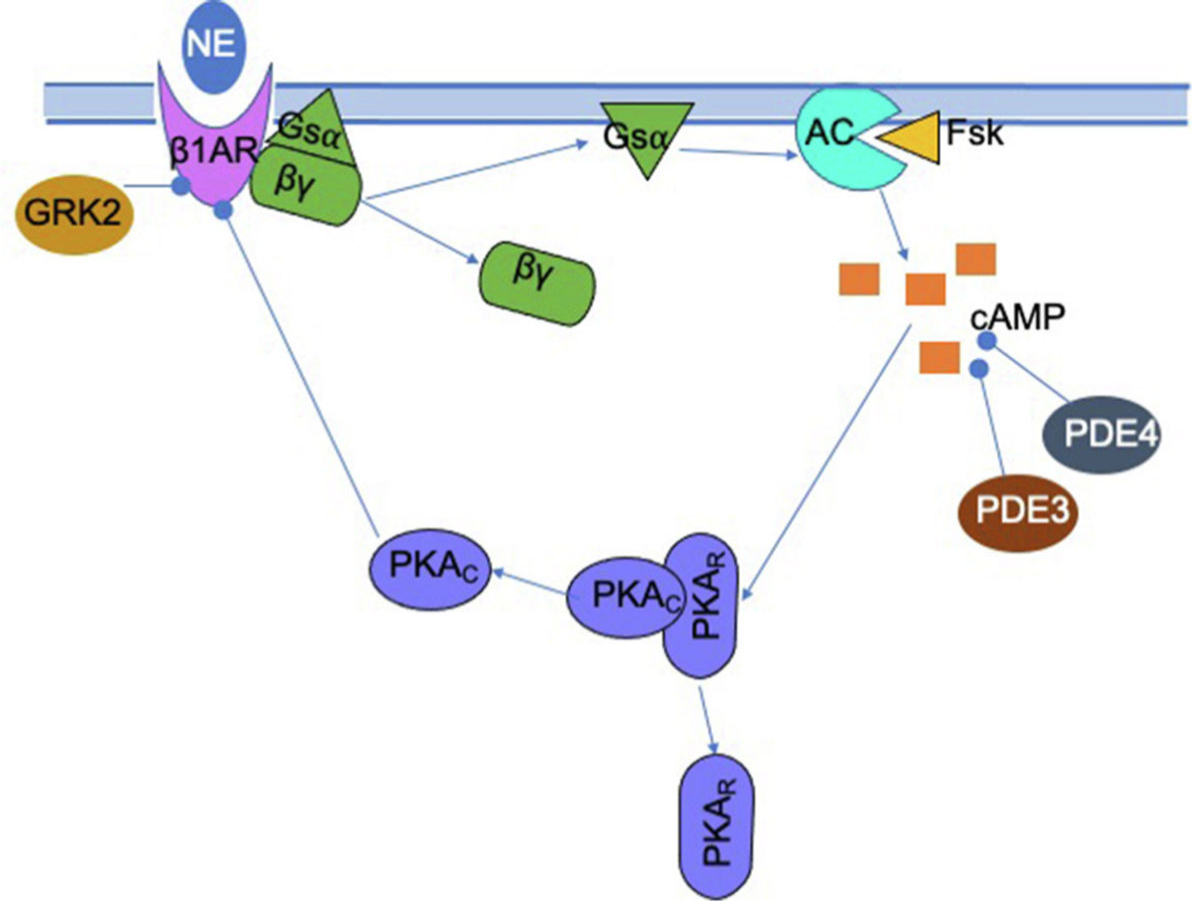
The release of norepinephrine activates a biochemical signaling pathway that results in intracellular physiological changes in a cardiac myocyte. The adrenergic agonist binds to a *β*_1_-AR, which activates the *Gs*_*α*_ subunit to stimulate adenylyl cyclases V and VI (AC), which then produces cAMP. cAMP activates PKA, the catalytic subunit (PKA_*C*_) of which phosphorylates numerous targets including the *β*_1_-AR, potassium channels, calcium channels, ryanodine receptors, phospholamban, and troponin I. Meanwhile, cAMP is degraded by phosphodiesterase 3 and 4 (PDE3, PDE4).

Cardiac modulation by the SNS occurs via the release of epinephrine and norepinephrine (NE) from sympathetic neurons directly onto cardiac myocytes expressing *β*-adrenergic receptors (*β*-ARs). The heart is densely innervated, such that individual cardiac cells in both the conduction pathway and the myocardium receive synaptic input from the SNS (Zaglia and Mongillo, 2017; Freeman et al., 2014). Cardiac cells predominantly express *β*_1_-adrenergic receptors (*β*_1_-ARs), which bind synaptic NE or adrenergic agonist and induce changes to electrophysiology and contractility. The activation of *β*_1_-ARs modulates an intracellular signaling pathway that, when activated, stimulates adenylyl cyclases 5 and 6 to increase production of cyclic AMP (cAMP), releasing the catalytic subunit of protein kinase A (PKA) to phosphorylate cellular targets (Fig. 1). Activated PKA phosphorylates delayed rectifier potassium channels *I*_*Kr*_ and *I*_*Ks*_, L-type calcium channels, and troponin I, as well as both ligand-bound and unbound *β*_1_-ARs, which are desensitized by phosphorylation. Ligand-bound receptors are also selectively phosphorylated and desensitized by G-protein coupled receptor kinase 2 (GRK2).

Although the mechanisms for sympathetic-induced arrhythmias are not fully understood, it is known that the effects of SNS activity are largely mediated through changes at a cellular scale, which occur via changes to the concentration of cAMP in individual myocytes. In single cells, *β*_1_-adrenergic activity can increase the propensity for arrhythmias by various means: enhancement of late sodium current or L-type calcium current increases the risk of EADs (Clancy et al., 1999; Weiss et al., 2010), especially in long-QT syndrome (LQTS) in cells with *I*_*Ks*_ block or *I*_*Kr*_/*I*_*Ks*_ mismatch (Banyasz et al., 2014; Schwartz et al., 2001; Vincent et al., 2009), while increased calcium influx increases the propensity for DADs (Bers, 2008). At the scale of the organ, the cellular changes induced by *β*_1_-AR activation can be arrhythmogenic in various pathologies including LQTS, myocardial infarction, atrial fibrillation, and heart failure (Grandi and Ripplinger, 2019), and it is estimated that roughly 50% of sudden deaths in heart failure are due to electrophysiological aberrations (Janse, 2004; Coronel et al., 2013). Since each of these arrhythmogenic processes depends on the excess production of cAMP via *β*-adrenergic signaling, it is essential to decipher the key dynamical mechanisms of the kinetics of the *β*_1_-AR biochemical cascade.

To elucidate the dynamics of cAMP in cardiac cells, it is necessary to analyze the kinetics of the transduction of a sympathetic stimulus from *β*_1_-AR activation to the increased production of cAMP and resulting active PKA. Upon sudden and prolonged activation of *β*_1_-adrenergic receptors, as occurs during a sympathetic surge, cAMP increases over one to two minutes and then gradually decreases to an intermediate level (Saucerman et al., 2004); the maximum achieved during the transient rise is often markedly higher than the final steady state. We refer to this transient cAMP over-elevation during the initial phase of the stimulus as “overshoot”. It is of interest to identify biological parameters that modulate overshoot, and how these parameters may be manipulated to change the amplitude of the large overshoots in cAMP concentration while maintaining the functional dynamic range of physiological output – that is, the range of attainable steady state cellular concentrations of cAMP. Mathematical modeling is a useful tool to analyze the mechanisms responsible for the temporal complexity of cAMP concentration in cardiac myocytes and to explore the changes in parameters that influence the dynamics of this behavior. Of the biophysically detailed computational models for the adrenergic signaling cascade in myocytes that have been constructed, perhaps most widely used is the Soltis-Saucerman model (Saucerman et al., 2003, 2004; Soltis and Saucerman, 2010), which connects *β*-AR signaling with electrophysiology in rabbit ventricular myocytes, including the processes outlined above and depicted in Fig. 1.

The present work uses the Soltis-Saucerman model as a foundation, owing to its biophysical detail, and aims to simplify this model to identify the rate-determining processes for the kinetics of the adrenergic signaling pathway. We use dimension reduction techniques to reduce the signaling subsystem of the original model to a two-dimensional system of differential equations. We then use phase plane techniques to analyze the mechanisms of overshoot in cAMP concentration and to identify ways to modify the amplitude of the overshoot, as well as to clarify the general relationships between parameters and outcomes of cAMP production across a range of conditions. Finally, we note that GRK2 is both known to interact with a variety of targets (Penela et al., 2010) and associated with cardiac pathology (Madamanchi, 2007). It is not known whether the effects of GRK2 overexpression and inhibition are mediated by adrenergic signaling or by other targets. Given that downregulation of GRK2 has been proposed as a synergistic therapy alongside *β*-blockers (Najafi et al., 2016; Cannavo et al., 2013), we consider the potential mechanisms by which changes to GRK2 activity might impact cellular cAMP signaling. Analysis of these mechanisms in the two-variable model using the phase plane elucidates how concurrent GRK2 inhibition might modify the effects of *β*-blockers on myocytes. Our results suggest that a simplified two-dimensional model can capture the extent to which the isolated adrenergic signaling pathway mediates both the potentially harmful effects of GRK2 in heart failure and the therapeutic benefits of its downregulation by pharmaceutical agents.

## 2. Model of *β*_1_-adrenergic signaling pathway

The Soltis-Saucerman model for electrophysiology (Saucerman et al., 2003, 2004; Soltis and Saucerman, 2010) uses a system of mass-action-based differential equations to simulate the processes of electrophysiology, calcium flux, and signaling from CaMKII and the sympathetic nervous system in a rabbit ventricular myocyte. We isolate the *β*-adrenergic signaling subsystem of the Soltis-Saucerman model, which stands alone and does not receive feedback from the downstream cellular targets or other model components.

The adrenergic signaling portion of the Soltis-Saucerman model contains sixteen variables (see Appendix A.1) that model the sequence of biochemical reactions triggered by the binding of norepinephrine (NE) or an adrenergic agonist to a *β*_1_-adrenergic receptor and lead to the activation of PKA. Seven of the variables are governed by differential equations. Of these seven dynamic variables, two variables are “read-out” components that do not affect other variables; two other variables can be removed using the conservation conditions, and a fifth variable can be removed by exploiting separation of time scales and setting the variable to its quasi-steady state. The resulting reduce system has two dynamics variables: the concentration of cAMP (*c*) and the concentration of non-desensitized *β*_1_-AR (*β*). The algebraic equations for variables in pseudo-equilibrium are left unchanged. A detailed description of the model reduction in presented in Appendix A.2.

The two-dimensional system of differential equations that describes *β*_1_-adrenergic signaling is:

(1) $$\frac{d\beta }{dt}=p_{10}\left(\beta_{tot}-\beta \right)-p_{9}\beta PKA_{c_{1}}(c)-F_{1}\left(\beta ;L_{tot}\right)$$

$$\frac{dc}{dt}=\left(\frac{p_{15}p_{20}}{p_{15}+p_{23}}\right)AC_{b}\left(\beta ;L_{tot}\right)+\left(\frac{p_{15}p_{21}}{p_{26}\left(p_{15}+p_{23}\right)}\right)AC_{s}\left(\beta ;L_{tot}\right)\;\;-\left(\frac{p_{16}p_{28}c_{f}(c)}{p_{29}+c_{f}(c)}+\frac{p_{17}p_{30}c_{f}(c)}{p_{31}+c_{f}(c)}\right)$$

where *L*_*tot*_ is the total concentration of norepinephrine (NE) or adrenergic agonist; *β*_*tot*_ is the total concentration of *β*_1_-ARs; *F*_1_ is the rate of *β*_1_-AR desensitization by GRK2; *c*_*f*_ is the concentration of “free” cAMP (not bound to PKA); *p*_9_ is the rate constant of *β*_1_-AR desensitization by PKA; *p*_10_ is the resensitization rate of phosphorylated *β*_1_-AR; *p*_15_ is cellular ATP concentration; and *p*_16_; *p*_17_; *p*_20_; *p*_21_ and *p*_23_ are rate constants and saturation constants associated with production of cAMP by AC and degradation of cAMP by PDE3 and PDE4. Details of the functions PKA_*c*1_; *F*_1_; *AC*_*b*_; *AC*_2_ and *c*_*f*_ are provided in the appendix. Parameters were unchanged from the full model described in (Saucerman et al., 2003, 2004), except in specific cases described in Sections 4.3–4.5. Simulations were performed in MATLAB using ode15s, and algebraic equations were solved using the fsolve root-finding algorithm with appropriate initial conditions.

The reduced model captures the four dynamic processes that govern the temporal dynamics of *β*_1_-adrenergic signaling: (1) the terms $\left(\frac{p_{15}p_{20}}{p_{15}+p_{23}}\right)AC_{b}\left(\beta ;L_{tot}\right)$ and $\left(\frac{p_{15}p_{21}}{p_{26}\left(p_{15}+p_{23}\right)}\right)AC_{s}\left(\beta ;L_{\text{tot}}\right)$ model the rate of production of cAMP by adenylyl cyclases V and VI at a basal rate and a rate stimulated by $G_{s,\alpha }^{GIP}$ (2) the terms $\left(\frac{{\text{p}}_{16}{\text{p}}_{28}c_{f}(c)}{{\text{p}}_{29}+c_{f}(c)}\right)$ *and* $\left(\frac{p_{17}p_{30}c_{f}(c)}{p_{31}+c_{f}(c)}\right)$ model the rate of degradation of cAMP by phosphodiesterases 3 and 4; (3) the terms $p_{9}PKA_{c_{1}}(c)\beta$ and $F_{1}\left(\beta ;L_{\text{tot}}\right)$ model the rate of desensitization of *β*_1_-ARs by PKA and by GRK2, respectively; and (4) the term $p_{10}\left(\beta_{tot}-\beta \right)$ models the rate of resensitization of desensitized *β*_1_-ARs. Details of the functions *PKA*_*c*1_; *F*_1_; *AC*_*b*_; *AC*_2_ and *c*_*f*_ are provided in Appendix A.2.

**Fig. 2.**
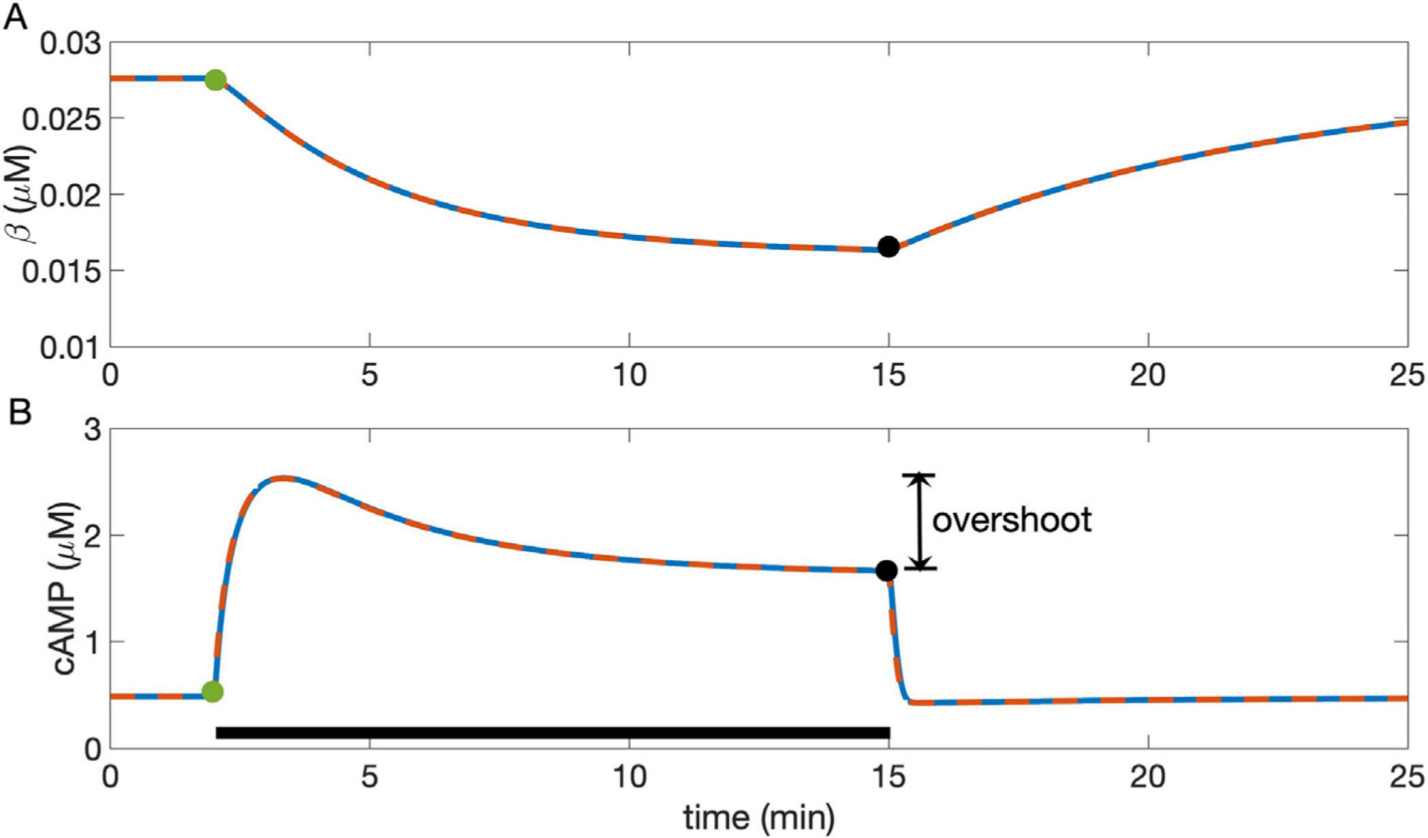
Predictions for cellular response to a change from basal conditions to 100 nM NE and subsequent return to 0 NE in the full (solid blue line) and reduced (dashed red line) Soltis-Saucerman model. Agonist is applied from 2 to 15 min of the simulation (black bar in B). A: both models predict a slow decrease in *β* upon application of NE, followed by a slow increase when NE is removed. B: in both models, cyclic AMP concentration transiently increases for 1–2 min and then gradually decays to a steady state in the presence of a high NE concentration. Overlay includes the trajectory from Fig. S1, panel A in Saucerman et al. (2004) and data (circles), taken from Hayes et al. (1980). Vertical double-arrow depicts “overshoot,” the difference between transient maximum and elevated steady state. Removal of NE leads to a small undershoot and return to the basal steady state. The models show nearly identical outputs, indicating that the reduction does not substantially change predictions. Green and black circles indicate steady-state values of variables for NE− and NE+ conditions, just preceding application and removal of NE respectively (see Fig. 3).

**Fig. A.2.**
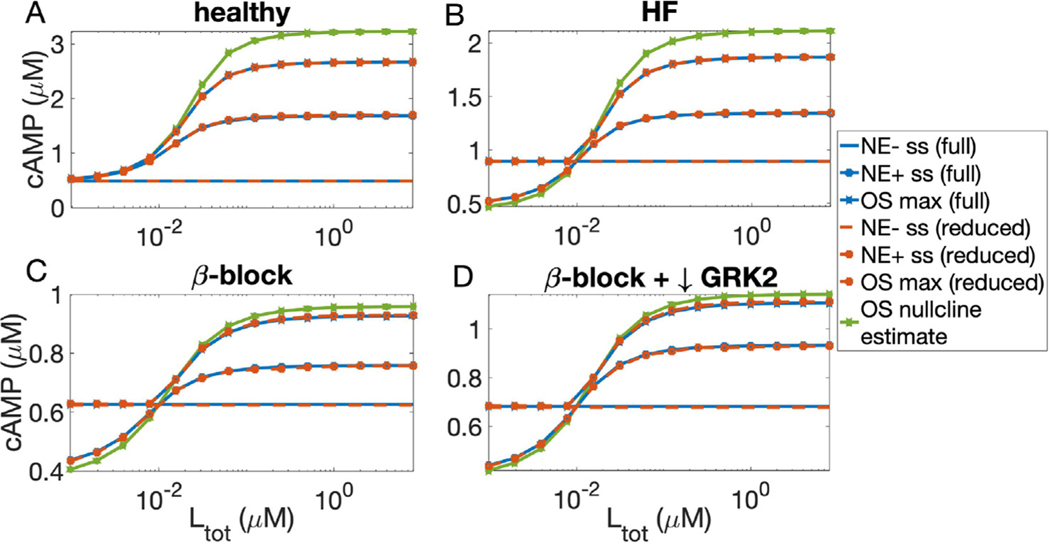
Comparison between the predictions of the full and reduced model across a range of *L*_*tot*_ values in each scenario described in Fig. 5. A: cAMP steady states with 0 *μ*M NE (NE- ss) and a nonzero NE concentration, and overshoot max (OS max) attained from an initial condition at the NE- ss, in the healthy condition (*β*_*tot*_ = 0.028 *μ*M, *k*_*GRK*2_ = 1.1*e* − 3 sec^−1^). B: as in A, but with NE- cAMP steady states computed using 0.01 *μ*M NE and *k*_*GRK*2_ = 2.2*e* − 3 sec^−1^. C: as in B, with *β*_*tot*_ = 0.014 *μ*M. D: as in C, with *k*_*GRK*2_ = 1.1*e* − 3. In all panels, the overshoot max in the full and reduced model are computed from simulations (red and blue curves) and compared with the estimate of overshoot max (green) computed using the value of the cAMP nullcline corresponding to the *β* concentration at the NE- steady state. The nullclines consistently overestimate the overshoot.

We validate the reduced model by comparing its predictions against those made by the full model for cAMP and non-desensitized *β*_1_-AR concentrations, as well as for concentrations of PKA and other components of the signaling pathway, under the abrupt application and removal of NE. As shown in the example in Fig. 2, the reduced model (red dashed curves) exhibits behavior almost indistinguishable from the full model (blue curves). This excellent agreement between the full model and the reduced model holds across a wide range of NE concentrations (0.001–10 μM) and parameter regimes (see Appendix A.3, Fig. A.2).

## 3. Results

### 3.1. Reduced model behavior

When norepinephrine or adrenergic agonist is added to a ligand-free system (Fig. 2), the concentration of non-desensitized *β*_1_-ARs gradually decays over tens of minutes (Fig. 2A). Cellular concentration of cyclic AMP increases over a period of approximately 1 min, reaching a transient maximum, and then gradually decreases to an intermediate value between the ligand-free resting state and the maximal concentration (Fig. 2B). In particular, Fig. 2 depicts the “overshoot” phenomenon that occurs when the initial condition is the steady state for the ligand-free system, and a high dose of 100 nM NE is added.

### 3.2. Phase plane analysis

We further examine the underlying mechanisms for the dynamics of the system by using the phase plane, which divides state space into regions where the variables each increase and decrease. The curves or “nullclines” delineating these regions are the zero contours for the derivatives of each dynamic variable. The resulting half-planes on either side of each nullcline form the regions of increase and decrease for each variable; the full plot is called a phase plane.

**Fig. 3.**
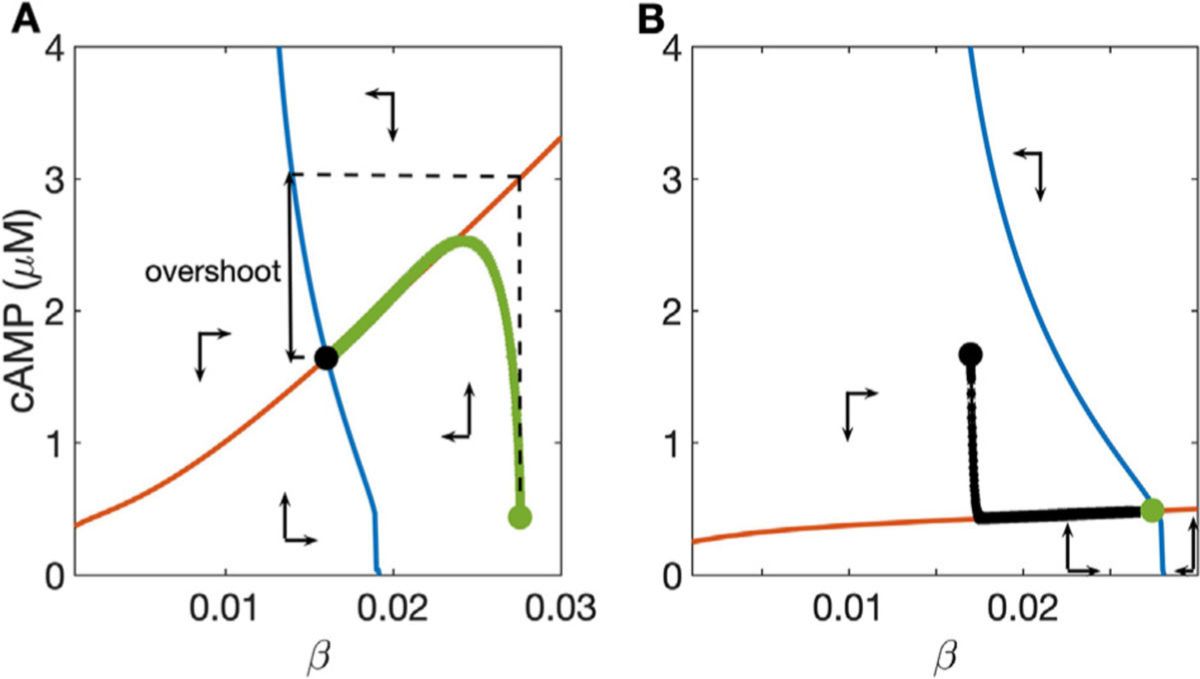
Phase plane for the two-variable reduced model: cAMP nullcline (red) and *β* nullcline (blue) divide state space into regions where cAMP concentration and active *β*_1_-AR concentration increase and decrease (see text). A: high dose, 100 nM NE; B: NE-free condition. Synaptic NE concentration changes the slope of the cAMP nullcline and the position of the *β* nullcline. Green and black circles are located at the steady states for the NE-free and high-dose NE conditions, respectively, and used as initial data for the alternate condition, producing the trajectories corresponding to the solutions shown in Fig. 2. Vertical arrow depicts the “overshoot,” in which the nearly vertical rise to the cAMP nullcline precedes a slower decay to the NE+ steady state. Note that cAMP concentration changes more rapidly than does the *β* concentration, leading to overshoot when the cAMP nullcline moves abruptly. The amplitude of the overshoot can be estimated by the height of the cAMP nullcline at the NE- steady state (i.e., vertical distance between green circle and red curve in A). The vertical difference between NE+ and NE− steady states (green and black circles) represents “dynamic range”, i.e. cellular responsiveness to ligand.

Fig. 3 shows the phase plane for the reduced signaling model (1) both in the NE-free condition (NE−) and in the presence of high NE or adrenergic agonist concentration (NE+). With no agonist (Fig. 3B), the cAMP nullcline is nearly horizontal, while the *β* nullcline is approximately vertical, and there is one stable steady state at their intersection. The cAMP variable *c* changes more rapidly than does concentration of non-desensitized *β*_1_-ARs, so that the system initialized away from the steady state reaches the *c* nullcline first before slowly tracing this nullcline to the global steady state. This transition is depicted by the green trajectory in Fig. 3A.

The cAMP and *β*_1_-AR nullclines shift in response to changes to the ligand concentration. When agonist concentration increases suddenly from 0 to 100 nM, as in the transition from Fig. 3B to A, the slope of the cAMP nullcline increases and the *β*_1_-AR nullcline moves to the left in the phase plane. Because *c* changes much more rapidly than *β*, the state of the system first moves almost vertically in the phase plane towards the cAMP nullcline, then traces this nullcline downward to the new global steady state as b adjusts more slowly (green curve in Fig. 3A). Note that the steepness of the cAMP nullcline under high-NE conditions paired with the difference in time scales between the dynamics of *c* and *β* creates the cAMP “overshoot” as the system evolves from the initial condition at the NE-steady state to the new at NE+ steady state. The magnitude of overshoot can be estimated by the vertical difference in (*β*, *c*) state space between the NE- steady state and the corresponding point on the cAMP nullcline in the NE+ condition (vertical difference between green circle and red curve in Fig. 3A). Note that this metric consistently overestimates the magnitude of the overshoot, but it enables a direct, mechanistic analysis of the relationships between parameters and cAMP dynamics, and it can provide an efficient approximation over a broad range of parameter conditions (e.g., the maximal error of the approximation in the result presented below is 20%).

The “dynamic range” of the *β*_1_-AR signaling pathway can be defined as the difference between the steady state cAMP concentration with no NE and the steady state with a high dose of NE. Under default parameter conditions, the dynamic range of cAMP is approximately 1 *μ*M (difference in cAMP between green and black circles in Fig. 3A and B). This range measures the overall responsiveness of the cell to adrenergic input. Moreover, this measure provides a relative estimate of the responsiveness of overall cardiac response to sympathetic tone, as heart rate increases with cAMP concentration.

### 3.3. Norepinephrine and phosphodiesterase modulate cAMP overshoot

Phase plane analysis can be used to efficiently quantify the relationship between cellular conditions and predicted outcomes, and to directly show how these outcomes depend on the steady-state relationships between the two variables. We selected parameters important to the four dynamic processes that affect the two-variable model: total phosphodiesterase concentration, total *β*_1_-AR concentration, and *β*_1_-AR GRK2 desensitization rate constant (*k*_*GRK*2_). As in Section 3.2, we approximated overshoot as the vertical difference in (*β, c*) state space between the NE- steady state and the corresponding point on the cAMP nullcline in the NE+ condition (e.g. vertical difference between green circle and red curve in Fig. 3A).

**Fig. 4.**
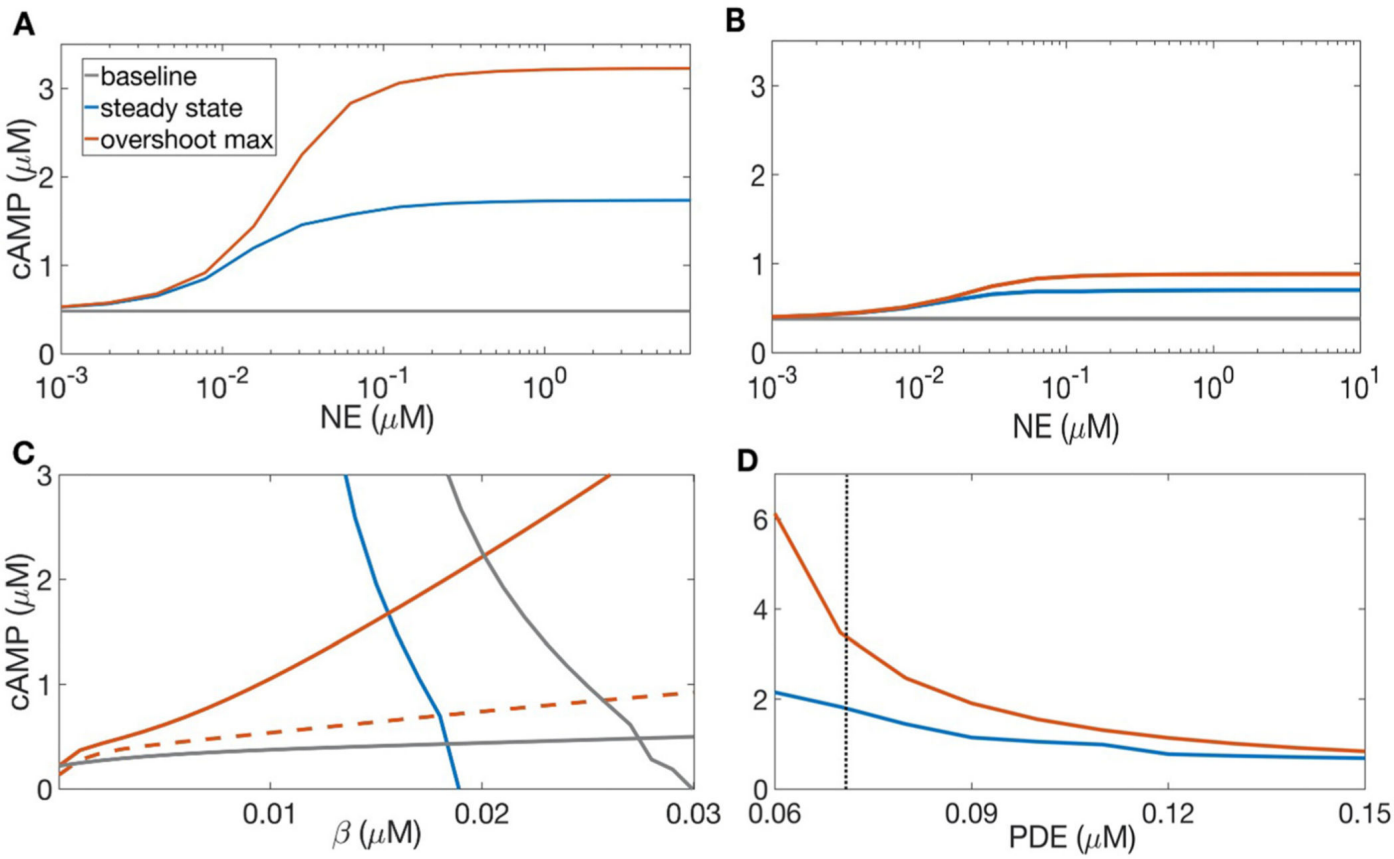
Effects of phosphodiesterase bulk concentration on cAMP overshoot. A: cAMP steady state (blue) and maximum (red), compared to the NE- steady state (gray), with total PDE concentration 0.072 *μ*M as in (Saucerman et al., 2003, 2004). As NE increases through several orders of magnitude, overshoot amplitude increases most sharply between 10 and 100 nM NE. B: cAMP steady state and maximal concentration, as in A, with bulk PDE concentration doubled to 0.144 *μ*M. The steady state and maximal concentrations of cAMP are both reduced. C: *c* and *β* nullclines with 1*μ*M NE, and total PDE concentrations 0.072 *μ*M (solid red curve) and 0.144 *μ*M (dashed red curve). The gray curves denote the NE− nullclines, and the blue curve depicts the NE+ *β* nullcline, which is unaltered by increased PDE. Increased concentration of PDE reduces the slope of the cAMP nullcline, changing the steady state concentrations of both *c* and *β* and the amplitude of the cAMP overshoot. D: cAMP steady state (blue) and maximum (red) for 1*μ*M NE, with varying concentrations of total phosphodiesterase (sum of PDE3 and PDE4). As PDE concentration increases over a narrow range of values, the amplitude of cAMP overshoot decreases.

As shown in Fig. 4A, with default parameters, overshoot amplitude increases markedly with NE concentration up to [NE] ≈ 100 nM, beyond which both the maximum and steady-state cAMP concentration saturate with respect to NE concentration (Fig. 4A). The dynamics of cAMP concentration are modulated by phosphodiesterases 3 and 4, depicted in Fig. 4B–D. Default PDE bulk concentration was taken to be 0.072 *μ*M, as in (Saucerman et al., 2003, 2004). Increased PDE concentration (0.144 *μ*M) reduces the slope of the high-NE cAMP nullcline (Fig. 4C). This diminishes the amplitude of cAMP overshoot, as the maximal cAMP concentration reached in the NE+ case is close to the steady state for the NE- case. Moreover, increased PDE concentration reduces the steady states and maximal cAMP concentrations over a broad range of NE concentrations, including at very high NE (Fig. 4B). Thus, the dynamic range of cAMP concentration, and therefore the responsiveness of the cell to a range of adrenergic input, is markedly diminished when PDE concentration is increased. This reduction in both overshoot amplitude and dynamic range of cAMP concentration takes place over a narrow range of total PDE concentrations (Fig. 4D).

### 3.4. Phase plane analysis of early heart failure and β-blockers

**Fig. 5.**
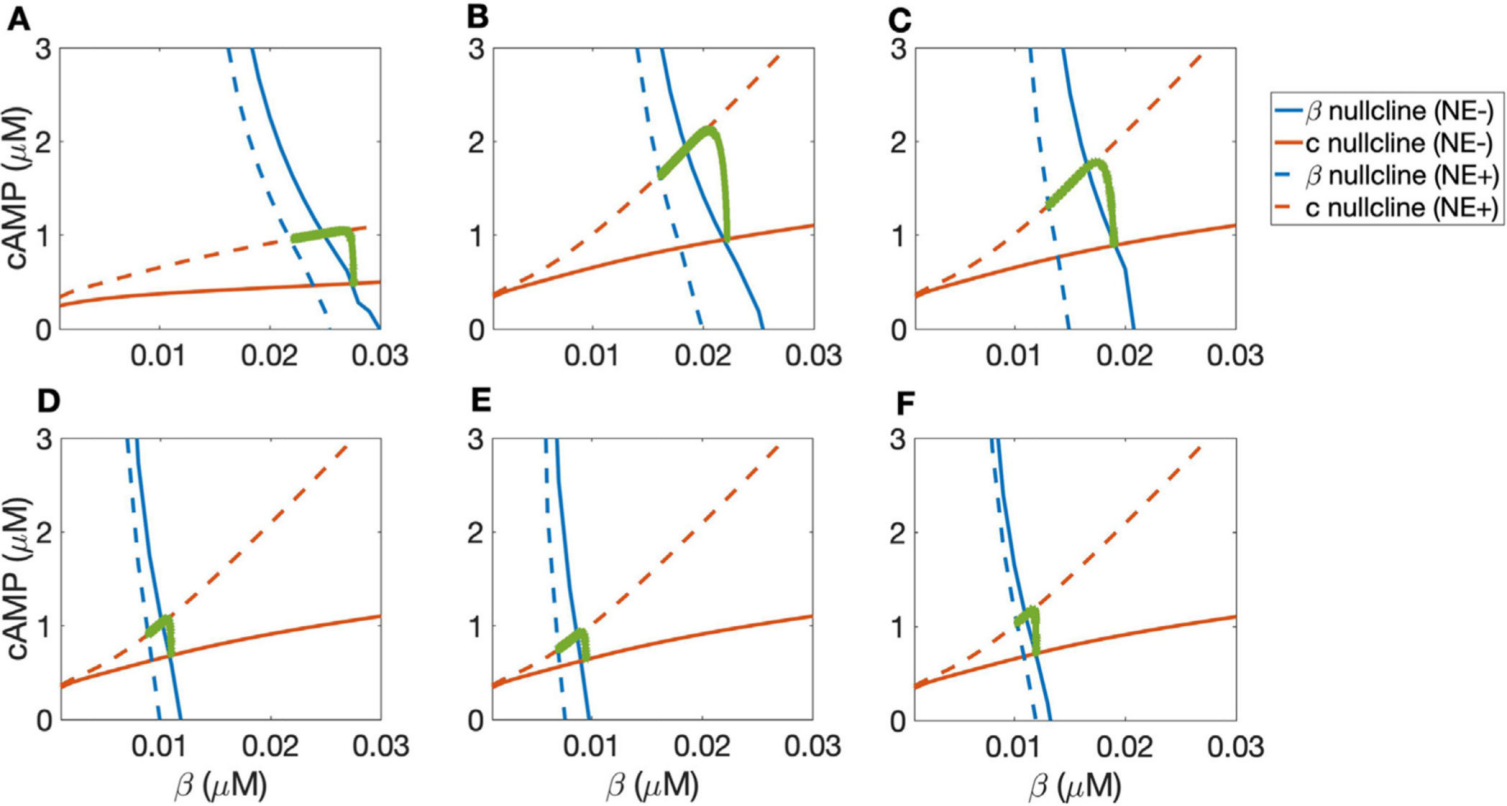
Putative effects of *β*-blocker treatment on markers of early heart failure. In all panels, blue curves represent *β* nullclines and red curves are *c* nullclines. Solid lines represent 0 NE (NE−) while dashed lines indicate the NE+ cases with varying concentrations of NE. Green curves are trajectories from simulations with initial condition at the NE− steady state transitioning to the NE+ steady state. A: phase plane with 0 and 10 nM NE and default parameters, corresponding to a healthy system. B: phase plane with cAMP and *β* nullclines for 10 and 100 nM NE, corresponding to elevated catecholamine levels at rest as in early heart failure. C: nullclines for 10 and 100 nM NE, as in early heart failure; additionally, *k*_*GRK*2_ is increased by a factor of 2, corresponding to up-regulation of GRK2. D: phase plane for 10 and 100 nM NE with total *β*_1_-AR concentration reduced by half and other parameters at default values. E: phase plane with 10 and 100 nM NE, with *β*_1_-AR concentration halved and *k*_*GRK*2_ doubled. F: phase plane with 10 and 100 nM NE with both *β*_1_-AR concentration and *k*_*GRK*2_ halved.

The relationships between various parameters and the phase plane can be used to investigate changes to cellular signaling akin to those that occur early in heart failure, which is associated with both chronic elevation of resting catecholamines and enhanced activity of GRK2. While *β*-blockers competitively inhibit *β*_1_-ARs, GRK2 has recently been proposed as an additional therapeutic target in heart failure (Cannavo et al., 2013). In Fig. 5, we demonstrate how cellular conditions associated with *β*_1_AR inhibition and GRK2 downregulation impact the adrenergic signaling system.

In Fig. 5, we examine the separate and joint effects of heart failure described above in Fig. 5. In all panels, blue curves represent *β* nullclines and red curves are *c* nullclines. Solid lines represent the 0 or “low” NE case (NE−) while dashed lines indicate the “high” NE (NE+) cases. Green curves represent trajectories of transition from the NE− steady state to the NE+ steady state, indicative of the cAMP response when NE is applied suddenly.

First, we assess the consequence of changes to baseline NE levels in early HF by changing the “low concentration” of ligand to 10 nM NE rather than 0, and considering the difference between a relatively high dose of 10 nM NE in a healthy condition with an increased high dose of 100 nM in heart failure (compare Fig. 5A and B). Higher *β*_1_-AR activity increases the slope of the cAMP nullcline, which increases the amplitude of the transient “overshoot” in cAMP concentration.

In Fig. 5C we plot the nullclines for 10 and 100 nM NE, as in B, and additionally increase *k*_*GRK*2_ by a factor of 2, corresponding to up-regulation of GRK2. The up-regulation of GRK2 shifts the NE+ *β*-nullcline (dashed blue curve) to the left, reducing the dynamic range of cAMP concentration. The phase plane in each of these cases depicts a markedly larger transient increase in cAMP production than is observed in the “healthy” case (contrast Fig. 5 B and C with A) due to the steeper cAMP nullcline, due to the elevated levels of NE both at rest and in heightened SNS activity. Meanwhile the high-NE *β* nullcline shifts to the left due to the increased GRK2 activity (Fig. 5B compared with C), reducing the difference between the NE− and NE+ steady state cAMP concentrations. These changes act to increase the overshoot amplitude and decrease the dynamic range. That is, they increase the high cAMP concentrations but reduce the overall responsiveness of the cell to adrenergic input.

In Fig. 5D–F, we consider the pharmacological effects of *β*-blockade and downregulation of GRK2 by varying the available *β*_1_-AR concentration *β*_*tot*_ and the GRK2 desensitization rate constant *k*_*GRK*2_. Panel D shows the phase plane for 10 and 100 nM NE with total *β*_1_-AR concentration reduced by half and other parameters at default values. Reducing total *β*_1_-AR concentration mimics inhibition of *β*_1_-ARs. Comparison between panels B and D shows the role of *β*_1_-AR availability alone in determining cellular cAMP dynamics. Comparison between C and D considers the joint effect of *β*_1_-AR inhibition and GRK2 inhibition in reducing *β*_1_-AR availability and reversing the up-regulation of GRK2. Panel E displays the phase plane with 10 and 100 nM NE, with *β*_1_-AR concentration halved and *k*_*GRK*2_ doubled. Comparison between C and E can be thought of as the scenario in which GRK2 activity increases in early heart failure, and *β*-blockers do not reduce this upregulation. Finally, in Fig. 5F we show the phase plane with 10 and 100 nM NE with both *β*_1_-AR concentration and *k*_*GRK*2_ halved. Comparison between C and F represents the case where *β*-blockers reduce *β*_1_-AR availability and inhibit GRK2, compensating for *k*_*GRK*2_ upregulation in early heart failure. In contrast to the “heart failure” conditions, the decrease in available *β*_1_-AR concentration shifts both NE− and NE+ *β*-nullclines to the left (Fig. 5, compare B and D), while the inhibition of GRK2 shifts the NE+ *β*-nullcline to the right, closer to the NE- *β* nullcline (Fig. 5, D–F). These changes jointly counteract the two simulated effects of early heart failure, reducing the amplitude of cAMP overshoot while partially restoring the dynamic range of cAMP concentration.

The important implication of the findings in Fig. 5 is that the two pathways of *β*-block and GRK2 downregulation counteract each other to move the NE− *β*-nullcline to the left and the NE+ *β* nullcline to the right, closer to the NE− *β*-nullcline, thereby controlling the amplitude of the overshoot while maintaining a portion of the dynamic range.

### 3.5. Quantifying overshoot and dynamic range in HF and treatment with β-blockers

**Fig. 6.**
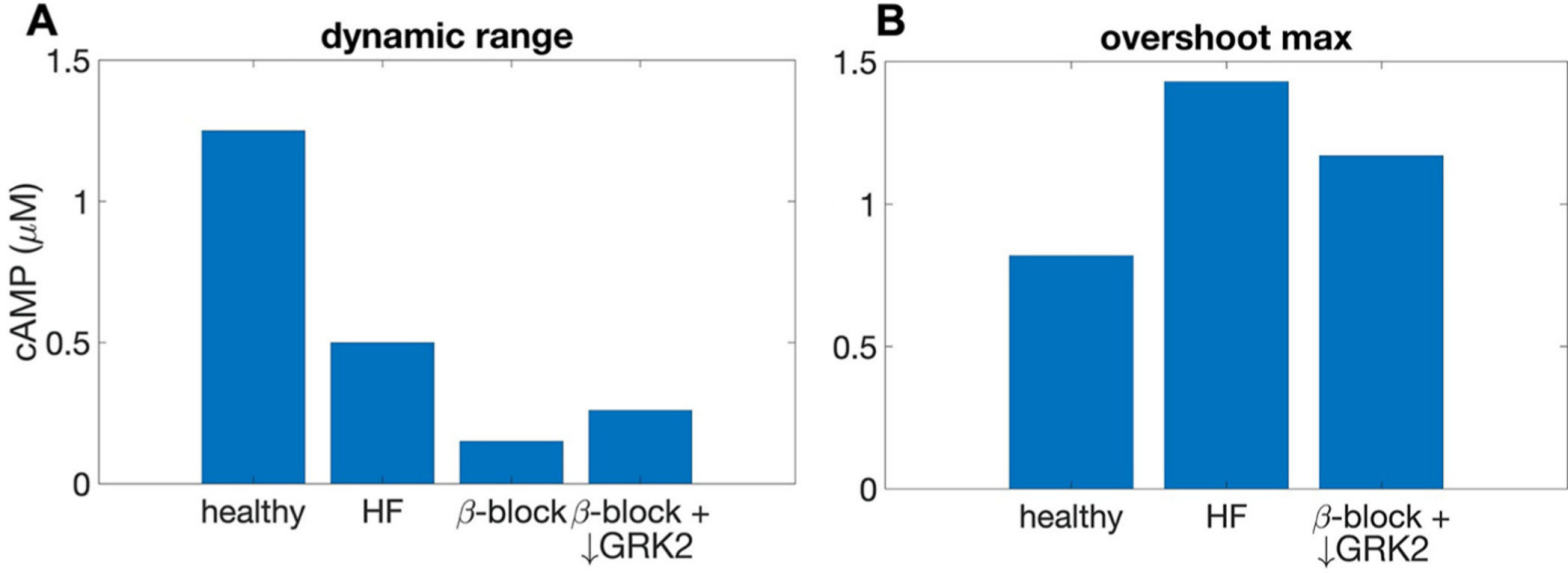
Effects of early heart failure and *β*_1_-AR inhibition on cellular cAMP baseline, maximum concentration attained during overshoot, and dynamic range as predicted by nullcline analysis. A: dynamic range in four conditions: healthy (baseline 0 *μ*M NE and “high dose” 10 *μ*M NE; early heart failure (elevated baseline NE [10*μ*M] and elevated “high dose” NE [100 *μ*M]; *β*-block (as in HF, and with total concentration of *β*-ARs reduced by 50%); and *β*-block with concurrent GRK2 downregulation (as in *β*-block, and with *k*_*GRK*2_ reduced by 50%). B: maximal cAMP concentration attained during overshoot for the amount of NE required to achieve a dynamic range of 0.26μM cAMP, compared across the “healthy”, “HF”, and “*β*-block + ↓ *GRK*2” scenarios.

The phase plane predicts that the two proposed mechanisms of pharmaceutical treatment, inhibition of *β*_1_-ARs and downregulation of GRK2, act synergistically to reduce the overshoot amplitude of cAMP while maintaining cellular responsiveness to changes in adrenergic agonist concentration. In Fig. 6, we quantify these effects using predictions generated by nullcline analysis.

Fig. 6A compares the total accessible dynamic range in four conditions. We simulate the elevated catecholamine levels in early heart failure by raising the “low dose” (NE−) of NE from 0 to 10 *μ*M and the “high dose” (NE+) from 10 to 100 *μ*M in the “heart failure” model. Upregulation of GRK2 is modeled by doubling the rate constant k_GRK2_ associated with GRK2 phosphorylation of the *β*_1_-AR. Together, elevated NE and reduced *k*_*GRK*2_ act to reduce dynamic range in the “heart failure” case compared to the “healthy” system. We simulate the action of selective *β*-blockers by reducing the total number of *β*_1_ARs by half, which further reduces the dynamic range. We simulate the additional effect of down-regulation of GRK2 concurrent with *β*_1_-AR inhibition by reducing *k*_*GRK*2_ to its baseline level, which recovers a portion of the dynamic range.

Fig. 6B compares the transient maximal cAMP concentration across parameter regimes for fixed values of dynamic range. Specifically, Fig. 6B shows the maximal cAMP concentration attained in each scenario during overshoot for the concentration of NE required to achieve a difference of 0.26 *μ*M cAMP between NE− and NE+ steady states, corresponding to the dynamic range seen in the concomitant drug treatment condition (*β*-block + ↓ GRK2). Respective NE concentrations were 6.5 nM in the “healthy” case, 27 nM in the “HF” case, and 1 *μ*M in the “*β*-block + ↓ GRK2” case. The early heart failure model attains a higher “overshoot” cAMP concentration (1.4 *μ*M) than does the healthy model (0.82 *μ*M), due to the elevated baseline levels of NE and consequently further elevated “high dose” NE concentration. With NE levels corresponding to the elevated baseline used to simulate early heart failure, the joint treatment of *β*-blockers with GRK2 downregulation reduces the overshoot amplitude (1.18 *μ*M cAMP) for a fixed dynamic range of 0.26 *μ*M cAMP. These results suggest that GRK2 downregulation could act synergistically with *β*_1_-AR inhibition to maintain the cellular responsiveness to adrenergic activity while reducing the transiently high levels of cAMP.

## 4. Discussion

The *β*-adrenergic signaling pathway is a complex biochemical cascade that is triggered by the binding of norepinephrine to *β*-adrenergic receptor and leads to the modulation of intracellular cAMP and PKA concentration, which in turn precipitate a wide variety of downstream effects that alter cellular electrochemical behavior. The Soltis-Saucerman model (Saucerman et al., 2003, 2004) is a widely-used mathematical model that provides a detailed description of this signaling pathway, using seven dynamic variables and several auxiliary variables. By eliminating non-essential variables and using quasi-steady state approximations, we reduce the *β*_1_-adrenergic signaling component of the Soltis-Saucerman model to a system of two ordinary differential equations for cellular cAMP concentration and non-desensitized *β*_1_-AR concentration. The success of this reduced model in replicating predictions of the full model reveals the rate-determining steps for the kinetics of the portion of the *β*_1_-adrenergic signaling cascade up to PKA activation. Namely, production of cAMP by adenylyl cyclase and degradation of cAMP by phosphodiesterase are the primary determinants for the kinetics of the sympathetic-induced rise in cellular cAMP concentration, on a time scale of ∼1 min. The desensitization of *β*_1_-ARs by PKA and by GRK2, and subsequent resensitization of the receptors, control the time scale of the slow decline of cAMP concentration to steady state during prolonged norepinephrine exposure, which occurs over ∼10 min. Thus, the reduced Soltis-Saucerman model suggests that cAMP production and degradation and *β*_1_-AR desensitization and resensitization dictate the temporal kinetics of cAMP, and the associated changes in heart rate and contractility, in response to changes in SNS activity as in physical and emotional arousal.

The analysis presented here explicitly quantifies the relationship between synaptic adrenergic agonist concentration and magnitude of cellular response. Moreover, the phase plane makes apparent that the size of this overshoot is modulated by the steepness of the cAMP nullcline in (*β*, c) space, the position of the *β*_1_-AR nullcline, and the difference in time scales between relatively fast cAMP dynamics and relatively slow desensitization and resensitization of *β*_1_-ARs.

Given the time scale of tens of seconds between the onset of receptor activation and increased cAMP concentration, the cAMP overshoot requires a sufficiently abrupt increase in adrenergic agonist. As demonstrated in our results, a sudden large increase in agonist concentration leads to transiently high cAMP concentration, i.e., overshoot. However, in the hypothetical scenario in which norepinephrine were to be applied at a more gradual rate commensurate with the stimulated production of cAMP by adenylyl cyclase, the quasi-steady-state cAMP concentration would also shift gradually, preventing the initial rise in cAMP concentration seen in overshoot. Instead, cAMP would increase monotonically to the NE+ steady state. Thus, we expect slow increases in SNS activity to cause markedly lower transient cAMP amplitude as compared with rapid increases in SNS activity, i.e. sympathetic surges.

Our analysis generates predictions regarding how cellular cAMP overshoot will respond to various changes to cellular conditions. Changes to bulk PDE concentration, for instance, alter the amplitude of cAMP overshoot, but also greatly reduce the range of attainable steady-state cAMP concentrations, impairing the heart’s ability to respond to fluctuations in sympathetic tone. Thus, PDE in cardiac myocytes is likely not an effective pharmacological target in counteracting the cellular adaptations present in heart failure. It has recently been shown, however, that PDE2A in stellate ganglion neurons may be an effective target for reducing sympathetic hyperactivity (Liu et al., 2018).

Adrenergic surges are known to be especially arrhythmogenic in individuals with heart failure and other pathologies. The success of the reduced model at replicating the predictions made by the full Soltis-Saucerman model demonstrates that the concentration of two variables, cAMP and non-desensitized *β*_1_-AR, capture the overall dynamics of sympathetic signaling. Therefore, the effects of heart failure, and other diseases of the sympathetic nervous system, on components of the *β*_1_-adrenergic signaling pathway can be reduced to how disease alters the factors that directly impact cAMP production/degradation and *β*_1_-AR (de) phosphorylation. The key factors influencing cAMP production are AC activity and PDE3,4 activity, while those that directly impact *β*_1_-AR phosphorylation are GRK2 and PKA_I_ activity; thus, we expect these factors to be effective targets for pharmaceutical therapy in diseases affecting the cardiac nervous system. It should be noted that we do not address chronic heart failure, which results in a wide array of structural and biochemical changes throughout the heart, including a reduction in *β*_1_-AR density as well as changes to sympathetic cardiac innervation (Ripplinger et al., 2016).

Our work demonstrates that the dynamics of only the cellular concentrations of cAMP and non-desensitized *β*_1_-AR can capture how two of the effects of early heart failure, alteration of the resting levels of adrenergic agonists and upregulation of GRK2, jointly act to change the maximal transient cAMP concentration and the longer-term overall responsiveness of the cell to sympathetic stimulation. Among the many structural and physiological changes in heart failure, up-regulation of GRK2 is frequently observed and suspected to play a role in cardiac pathology, whether due to its effects on adrenergic signaling or other interactions (Madamanchi, 2007). Increased levels of baseline norepinephrine increase the slope of the cAMP nullcline, which heightens the transient cAMP amplitude during “overshoot”. Meanwhile, the upregulation of GRK2 activity shifts the *β* nullcline to favor lower concentrations of *β*, reducing the cAMP concentration at the stimulated steady state and therefore diminishing the cell’s responsiveness to NE concentration changes. These results, while omitting the complexity of the compensatory mechanisms present in various stages of heart failure, provide a qualitative proof of concept demonstrating that the effects of early stages of heart failure on the adrenergic signaling pathway can be captured by the processes controlling the dynamics of cAMP and non-desensitized *β*_1_-adrenergic receptors.

Pharmacological inhibitors of *β*-adrenergic receptors, known as *β*-blockers, are generally considered protective in heart failure, but often also come with cardiac and other medical risk (Kotecha et al., 2017; Bohm and Maack, 2000; Cruickshank, 2000; Bouzamondo et al., 2003). Modeling work has considered the effects of *β*-blockers on both the “maintenance” and the “inhibition” of cellular responsiveness to adrenergic stimulation (Amanfu and Saucerman, 2014), suggesting that these two processes need not be viewed as mutually contradictory. Separately, recent work has identified a wide range of targets of GRK2 (Penela et al., 2010) and demonstrated that reduction of GRK2 activity, either genetic or pharmacologic, may improve overall cardiovascular function, particularly in heart failure (Schumacher et al., 2015; Lymperopoulos et al., 2013). Some experiments suggest that diminished GRK2 activity may act synergistically with *β*-blocker therapeutic drugs to prevent mortality risk in individuals with heart failure (Najafi et al., 2016; Schumacher et al., 2015; Lymperopoulos et al., 2013; Cannavo et al., 2013). Our results suggest a mechanistic justification for this hypothesis: lowering GRK2 activity alongside *β*_1_-AR inhibition reduces the amplitude of cAMP overshoot, and partially rescues the dynamic range of cAMP concentration compared with *β*-blockers alone in heart failure conditions. Taken together, these “maintenance” and “inhibition” processes work to counteract the simulated effects of early heart failure. To evaluate these predictions, our simulations should be compared with the efficacy of various *β*-blockers and GRK2 inhibitors, administered both separately and concurrently, for reducing mortality and heart failure symptoms particularly in early HF. While the predictions made by this model are qualitative rather than precise, the two-variable model provides a simple framework by which to assess and compare how various pharmaceutical treatments may affect adrenergic signaling.

It has been posited that an imbalance between the magnitude and timing of sympathetic and parasympathetic nervous system activity is a primary driver for arrhythmias in heart disease. Prior modeling work (Iancu et al., 2007, 2008) has explained a cellular cAMP “overshoot” phenomenon with similar temporal dynamics to that shown here as a consequence of this time-scale mismatch along with subcellular compartmentation of separate pools of cAMP and signaling components. By examining the isolated *G*_*s*_-mediated pathway, we have demonstrated that overshoot can occur and can be modified independent of parasympathetic input. Given the prior evidence that mismatch between sympathetic and parasympathetic activity enhances cAMP transient elevation and heightens arrhythmogenic risk, it would be beneficial to further explore the subcellular signaling pathways involved in these two systems, and the interaction between the two. For instance, experiments with cells containing the *β*_1_-adrenergic signaling machinery, but lacking muscarinic receptors or inhibitory G-protein, could differentiate between *β*_1_-AR desensitization and parasympathetic nervous system activity as mechanisms for cellular cAMP overshoot during sympathetic stimulation.

## Figures and Tables

**Fig. A.1.**
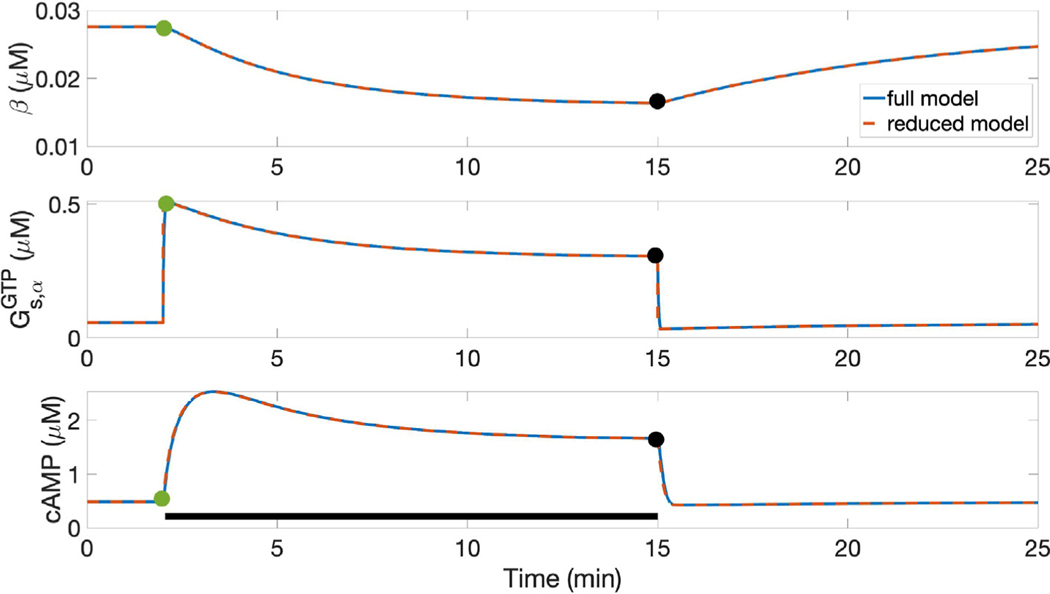
When the full model is initialized from a steady state with 0 NE, and 1 *μ*M NE is abruptly added, $G_{s,\alpha }^{\text{GTP}}$ adapts to its quasi-steady state value much more rapidly than do cAMP and *β*_1_*AR* concentrations. This justifies the simplification that $G_{s,\alpha }^{\text{GTP}}$ reaches quasi-steady state instantaneously, reducing the system of three variables to a two-dimensional system.

## Appendix A

### A.1. Soltis-Saucerman model

The adrenergic signaling subsystem of the original model (Saucerman et al., 2003, 2004, Soltis and Saucerman, 2010) has 16 variables (Table A.1):

The variables obey a system of ordinary differential equations (ODEs) and differential algebraic equations (DAEs), as follows. The algebraic equations reflect conservation laws and steady-state concentrations for reactions assumed to reach steady state instantaneously.

**Table A.1:** Variable names and definitions for the *β*_1_-adrenergic signaling subsystem of the Soltis-Saucerman model.

Variable number	Variable name	Description
1	*L*	*β*-AR agonist
2	*R*	*β*-AR
3	*G*	available *G*-protein
4	*β*	unphosphorylated *β*_1_-AR
5	*β_BARK_*	*β*-AR phosphorylated by *β*-ARK
6	*β_PKA_*	*β*-AR phosphorylated by PKA
7	*G_α, GTP_*	Total $G_{\alpha }^{\text{GTP}}$
8	*G_α, GDP_*	$G_{\alpha }^{\text{GDP}}$
9	$G_{\beta \gamma }$	$G_{\beta \gamma }$
10	$G_{\alpha ,\mathrm{GTP}}^{f}$	Free $G_{\alpha }^{\text{GTP}}$
11	Fsk	Forskolin
12	AC	adenylyl cyclase
15	*c*	total cAMP
16	*c_f_*	free cAMP
17	*PKA*_*c*_1__	Catalytic subunit of *PKA_I_*
18	*PKA*_*c*_2__	Catalytic subunit of *PKA_II_*

#### Algebraic Equations:

Ligand-Receptor Equations

$$L=L_{\text{tot}}-\frac{L\cdot R}{p_{4}}-\frac{L\cdot R\cdot G}{p_{4}\cdot p_{5}}$$

$$R=\beta -\frac{L\cdot R}{p_{4}}-\frac{L\cdot R\cdot G}{p_{4}\cdot p_{5}}-\frac{R\cdot G}{p_{6}}$$

$$G=p_{3}-\frac{L\cdot R\cdot G}{p_{4}\cdot p_{5}}-\frac{R\cdot G}{p_{6}}$$

G-protein-AC activation equations

$$G_{\alpha ,GTP}^{f}=G_{\alpha ,GTP}-\frac{G_{\alpha ,GTP}^{f}\cdot AC}{p_{26}}$$

$$AC=p_{14}-\frac{G_{\alpha ,GTP}^{f}\cdot AC}{p_{26}}$$

cAMP-PKA equations

$$c_{f}=c-\left[\frac{p_{36}}{c_{f}}\cdot \frac{PKA_{c_{1}}}{p_{38}}\cdot PKA_{c_{1}}\cdot \left(1+\frac{p_{35}}{p_{39}+PKA_{c_{1}}+PKA_{c_{2}}}\right)\right]$$

$$\;2\frac{PKA_{c_{1}}}{p_{38}}\cdot PKA_{c_{1}}\cdot \left(1+\frac{p_{35}}{p_{39}+PKA_{c_{1}}+PKA_{c_{2}}}\right)$$

$$\;-2PKA_{c_{1}}\cdot \left(1+\frac{p_{35}}{p_{39}+PKA_{c_{1}}+PKA_{c_{2}}}\right)$$

$$\;-\left[\frac{p_{36}}{c_{f}}\cdot \frac{PKA_{c_{2}}}{p_{38}}\cdot PKA_{c_{2}}\cdot \left(1+\frac{p_{35}}{p_{39}+PKA_{c_{1}}+PKA_{c_{2}}}\right)\right]$$

$$\;-2\frac{PKA_{c_{2}}}{p_{38}}\cdot PKA_{c_{2}}\cdot \left(1+\frac{p_{35}}{p_{39}+PKA_{c_{1}}+PKA_{c_{2}}}\right)$$

$$\;-2PKA_{c_{2}}\cdot \left(1+\frac{p_{35}}{p_{39}+PKA_{c_{1}}+PKA_{c_{2}}}\right)$$

$$0=2p_{33}c_{f}^{2}-PKA_{c_{1}}\left(1+\frac{p_{35}}{p_{39}+PKA_{c_{1}}+PKA_{c_{2}}}\right)$$

$$\;\times \left[\left(\frac{p_{36}\cdot p_{37}}{p_{38}}+\frac{p_{36}\cdot c_{f}}{p_{38}}+\frac{c_{f}^{2}}{p_{38}}\right)PKA_{c_{1}}+c_{f}^{2}\right]$$

$$0=2p_{34}c_{f}^{2}-PKA_{c_{2}}\left(1+\frac{p_{35}}{p_{39}+PKA_{c_{1}}+PKA_{c_{2}}}\right)$$

$$\;\times \left[\left(\frac{p_{36}\cdot p_{37}}{p_{38}}+\frac{p_{36}\cdot c_{f}}{p_{38}}+\frac{c_{f}^{2}}{p_{38}}\right)PKA_{c_{2}}+c_{f}^{2}\right]$$

#### Differential Equations:

*β*_1_ – AR dynamics

$$\frac{d\beta }{dt}=p_{8}\beta_{BARK}-\frac{p_{7}}{p_{4}}L\cdot R-\frac{p_{7}}{p_{4}\cdot p_{5}}L\cdot R\cdot G+p_{10}\beta_{PKA}-p_{9}\beta PKA_{c_{1}}$$

$$\frac{d\beta_{BARK}}{dt}=-p_{8}\beta_{BARK}+\frac{p_{7}}{p_{4}}L\cdot R+\frac{p_{7}}{p_{4}\cdot p_{5}}L\cdot R\cdot G$$

$$\frac{d\beta_{PKA}}{dt}=-p_{10}\beta_{PKA}+p_{9}\beta PKA_{c_{1}}$$

G-protein dynamics

$$\frac{dG_{\alpha ,GTP}}{dt}=\frac{p_{11}}{p_{6}}R\cdot G+\frac{p_{11}}{p_{4}\cdot p_{5}}L\cdot R\cdot G-p_{12}G_{\alpha ,GTP}$$

$$\frac{dG_{\alpha ,GDP}}{dt}=p_{12}G_{\alpha ,GTP}-p_{13}G_{\alpha ,GDP}G_{\beta \gamma }$$

$$\frac{dG_{\beta \gamma }}{dt}=\frac{p_{11}}{p_{6}}R\cdot G+\frac{p_{11}}{p_{4}\cdot p_{5}}L\cdot R\cdot G-p_{13}G_{\alpha ,GDP}G_{\beta \gamma }$$

cAMP dynamics

$$\frac{dc}{dt}=\left(\frac{p_{15}p_{20}}{p_{23}+p_{15}}\right)AC+\left(\frac{p_{15}p_{21}}{p_{24}+p_{15}}\right)G_{\alpha ,GTP}^{f}AC\;\;-\frac{p_{16}p_{28}c_{f}}{p_{29}+c_{f}}-\frac{p_{17}p_{30}c_{f}}{p_{31}+c_{f}}$$

### A.2. Reduction to two-variable model

STEP 1 (G-protein dynamics): Note that the variables *G*_*α,GDP*_ and *G*_*βγ*_ do not appear in any equations other than the differential equations governing their own dynamics. Therefore, the equations for *G*_*α,GDP*_ and *G*_*βγ*_ can be removed from the system, leaving only the single differential equation for *G*_*α,GDP*_ in the G-protein subsystem.

STEP 2 (*β*_1_-AR dynamics): Note that the three states of the *β*_1_-AR (*β*, *β*_*BARK*_, and *β*_*PKA*_) obey the conservation law

$$\beta +\beta_{\beta ARK}+\beta_{PKA}=\beta_{tot}$$

where the constant *β*_*tot*_ is the total concentration of *β*_1_-ARs. Furthermore, because *p*_8_ = *p*_10_ in the Soltis-Saucerman model, the variables *β*_*BARK*_ and *β*_*PKA*_ representing phosphorylated *β*_1_-AR states only appear as a sum in the differential equation for *β*. Therefore, *β*_*BARK*_ and *β*_*PKA*_ can be removed by replacing their sum with *β*_*tot*_ − *β*, which yields the single differential equation for the dynamics of *β*.

Thus, these observations yield a system of three differential equations for cAMP dynamics:

$$\frac{d\beta }{dt}=p_{10}\left(\beta_{tot-\beta }\right)-\frac{p_{7}}{p_{4}}L\cdot R-\frac{p_{7}}{p_{4}\cdot p_{5}}L\cdot R\cdot G-p_{9}\beta PKA_{c_{1}}$$

$$\frac{dG_{\alpha ,GTP}}{dt}=\frac{p_{11}}{p_{6}}R\cdot G+\frac{p_{11}}{p_{4}\cdot p_{5}}L\cdot R\cdot G-p_{12}G_{\alpha ,GTP}$$

$$\frac{dc}{dt}=\left(\frac{p_{15}p_{20}}{p_{23}+p_{15}}\right)AC\left(G_{\alpha ,GTP}\right)+\left(\frac{p_{15}p_{21}}{p_{24}+p_{15}}\right)G_{\alpha ,GTP}^{f}\cdot AC\left(G_{\alpha ,GTP}\right)\;\;-\frac{p_{16}p_{28}c_{f}}{p_{29}+c_{f}}-\frac{p_{17}p_{10}q_{f}}{p_{31}+c_{f}}$$

We define *F*_1_(*β*; *L*_*tot*_) as the desensitization rate of *β*_1_-AR phosphorylated by GRK2 (note that p_7_ = k_GRK2_):

$$F_{1}\left(\beta ;L_{\text{tot}}\right)=k_{\text{CRK2}}\left(\frac{1}{p_{4}}L\cdot R+\frac{1}{p_{4}\cdot p_{5}}L\cdot R\cdot G\right)$$

and *F*_2_(*β*; *L*_*tot*_) as the rate of G-protein activation:

$$F_{2}\left(\beta ;L_{tot}\right)=\frac{p_{11}}{p_{6}}R\cdot G+\frac{p_{11}}{p_{4}\cdot p_{5}}L\cdot R\cdot G$$

where L, R, and G are obtained by solving the LRG equations. (Note that *k*_*GRK2*_ is named as *k*_*βARKp*_ in Saucerman et al., 2004.) Both *AC*(*G*_*α,GTP*_) and $G_{\alpha ,GTP}^{f}$ can be obtained explicitly by solving the G-protein-AC algebraic equations:

$$AC=\frac{-G_{\alpha ,GTP}+p_{14}-p_{26}+\sqrt{{\left(p_{14}+p_{26}-G_{\alpha ,GTP}\right)}^{2}+4p_{26}G_{\alpha ,GTP}}}{2}$$

$$G_{\alpha ,GTP}^{f}=\frac{G_{\alpha ,GTP}-p_{14}+p_{26}+\sqrt{{\left(p_{14}+p_{26}-G_{\alpha ,GTP}\right)}^{2}+4p_{26}G_{\alpha ,GTP}}}{2}$$

and *PKA*_*c*1_(*c*) and *c*_*f*_ (*c*) are obtained by solving the cAMP-PKA algebraic equations.

This system of three differential equations can be further reduced to a system of two differential equations. Note that *G*_*α,GTP*_ changes much more rapidly than do *β* and *c* (see Fig. A.1). Therefore, we can exploit this separation of time scales and eliminate *G*_*α,GTP*_ by equilibrating to its quasi-steady value:

$$G_{\alpha ,GTP}=\frac{p_{11}}{p_{12}}\left(\frac{RG}{p_{6}}+\frac{LRG}{p_{4}p_{5}}\right)=\frac{F_{2}\left(\beta ;L_{\text{tot}}\right)}{p_{12}}$$

Thus, we obtain our reduced model (1):

$$\frac{d\beta }{dt}=p_{10}\left(\beta_{tot}-\beta \right)-p_{9}\beta PKA_{c_{1}}-F_{1}\left(\beta ;L_{tot}\right)$$

$$\frac{dc}{dt}=\left(\frac{p_{15}p_{20}}{p_{23}+p_{15}}\right)AC_{b}\left(\beta ;L_{tot}\right)+\left(\frac{p_{15}p_{21}}{p_{24}+p_{15}}\right)AC_{s}\left(\beta ;L_{tot}\right)\;\;-\frac{p_{16}p_{28}c_{f}}{p_{29}+c_{f}}-\frac{p_{17}p_{30}c_{f}}{p_{31}+c_{f}}$$

where $AC_{b}=AC,$, so that $\left(\frac{p_{15}p_{20}}{p_{23}+p_{15}}\right)AC_{b}\left(\beta ;L_{tot}\right)$ represents the rate of cAMP produced by adenylyl cyclase at a basal rate, not activated by $G_{\alpha ,GTP}$, and $AC_{s}=AC\cdot G_{\alpha ,GTP}^{f}$, so that $\left(\frac{p_{15}p_{21}}{p_{24}+p_{15}}\right)AC_{s}\left(\beta ;L_{tot}\right)$ represents the rate of cAMP produced by adenylyl cyclase stimulated by $G_{\alpha ,GTP}$.

### A.3. Validation of reduced model

We validated the reduced model by comparing its predictions to those made by the full model across a range of NE concentrations in each of the scenarios described in Fig. 5. In Fig. A.2A, we compare the cAMP steady states with 0 *μ*M NE (NE-ss) and a range of nonzero NE concentrations (NE+ss), and overshoot max (OS max) attained from an initial condition at the NE- steady state, in the healthy condition (*β*_*tot*_ = 0.028 *μ*M, *k*_*GRK*2_ = 1.1*e* − 3 sec^−1^). The reduced model replicates the predictions made by the full model for the steady states. As described in Fig. 3, the overshoot maximum is estimated for the reduced model using the point on the cAMP nullcline corresponding to the *β* value at the NE- steady state. In the full model, the overshoot maximum is computed using the maximum value of cAMP attained during the simulation. This results in a consistent overestimate of the overshoot maximum for the reduced model. In Fig. A.2B, we compute NE- cAMP steady states using 0.01 *μ*M NE, and increase *k*_*GRK*2_ to 2.2*e* − 3 sec^−1^, to simulate early heart failure. In Fig. A.2C we consider the effect of *β*-block by reducing *β*_*tot*_ to 0.014 *μ*M, with baseline NE and *k*_*GRK*2_ corresponding to the “HF” model. Finally, in Fig. A.2D we consider the joint effect of *β*-block with *k*_*GRK*2_ inhibition by reducing *k*_*GRK*2_ to its standard value of 1.1*e* − 3 sec^−1^, while keeping all other parameters fixed from Fig. A.2C. In all four situations, the reduced model closely captures the steady states predicted by the full model, and the overshoot calculation using the nullclines slightly overestimates the maximum cAMP concentration.
